# Speech-derived haptic stimulation enhances speech recognition in a multi-talker background

**DOI:** 10.1038/s41598-023-43644-3

**Published:** 2023-10-03

**Authors:** I. Sabina Răutu, Xavier De Tiège, Veikko Jousmäki, Mathieu Bourguignon, Julie Bertels

**Affiliations:** 1 Laboratoire de Neuroanatomie et de Neuroimagerie Translationnelles (LN2T), UNI – ULB Neuroscience Institute, Université Libre de Bruxelles (ULB), Brussels, Belgium; 2 Service de Neuroimagerie Translationnelle, Hôpital Universitaire de Bruxelles (H.U.B.), CUB Hôpital Erasme, Université Libre de Bruxelles (ULB), Brussels, Belgium; 3 Aalto Neuroimaging, Aalto University, Espoo, Finland; 4 BCBL, Basque Center on Cognition, Brain and Language, 20009 San Sebastián, Spain; 5 Laboratory of Neurophysiology and Movement Biomechanics, UNI – ULB Neuroscience Institute, Université Libre de Bruxelles (ULB), Brussels, Belgium; 6 ULBabylab, Center for Research in Cognition and Neurosciences (CRCN), UNI – ULB Neuroscience Institute, Université Libre de Bruxelles (ULB), Brussels, Belgium

**Keywords:** Psychology, Human behaviour, Sensory processing, Translational research

## Abstract

Speech understanding, while effortless in quiet conditions, is challenging in noisy environments. Previous studies have revealed that a feasible approach to supplement speech-in-noise (SiN) perception consists in presenting speech-derived signals as haptic input. In the current study, we investigated whether the presentation of a vibrotactile signal derived from the speech temporal envelope can improve SiN intelligibility in a multi-talker background for untrained, normal-hearing listeners. We also determined if vibrotactile sensitivity, evaluated using vibrotactile detection thresholds, modulates the extent of audio-tactile SiN improvement. In practice, we measured participants’ speech recognition in a multi-talker noise without (audio-only) and with (audio-tactile) concurrent vibrotactile stimulation delivered in three schemes: to the left or right palm, or to both. Averaged across the three stimulation delivery schemes, the vibrotactile stimulation led to a significant improvement of 0.41 dB in SiN recognition when compared to the audio-only condition. Notably, there were no significant differences observed between the improvements in these delivery schemes. In addition, audio-tactile SiN benefit was significantly predicted by participants’ vibrotactile threshold levels and unimodal (audio-only) SiN performance. The extent of the improvement afforded by speech-envelope-derived vibrotactile stimulation was in line with previously uncovered vibrotactile enhancements of SiN perception in untrained listeners with no known hearing impairment. Overall, these results highlight the potential of concurrent vibrotactile stimulation to improve SiN recognition, especially in individuals with poor SiN perception abilities, and tentatively more so with increasing tactile sensitivity. Moreover, they lend support to the multimodal accounts of speech perception and research on tactile speech aid devices.

## Introduction

Understanding what others are saying is a fundamental task for human communication. Yet, this is scarcely accomplished with complete ease due to the prevalence of background noise in our modern environments. Adverse auditory conditions hinder the neural processing of speech signals, resulting in reduced intelligibility and comprehension difficulties^1^. In such situations, the beneficial effect of non-auditory, visual cues, is well established, with listeners naturally leveraging visible lip movements to enhance speech-in-noise (SiN) understanding^2^. In the presence of multiple talkers (i.e., “*cocktail party*” settings) visual speech information has been found to provide significant improvements in speech recognition of up to 4.6 dB compared to auditory-only conditions^3^ and to enhance word recognition performance by 7%^4^. The extent to which speech cues transmitted through other senses can afford a similar benefit in multi-talker noise conditions is, however, less documented.

Several studies have demonstrated the potential of speech information presented haptically, i.e., through the tactile sense, to act as an adjuvant to SiN perception. Speech-derived haptic stimuli delivered to either the hand or wrists have been found to enhance the perception of syllables, words, and even full sentences^5–10^. The extent of this improvement is highly variable, ranging from 1 dB^10^ up to 10 dB^9^, and has been demonstrated in both hearing-impaired^11,12^ and normal-hearing listeners, even without^5–7^ or with some^8,9^ training. For the majority of these studies^6–10^, the supplemental haptic input consisted in vibrotactile stimulation. This type of input is the best candidate to haptically enhance SiN perception, since vibrotactile signals can be designed to fully correspond to the characteristic sensorial events of the auditory modality, i.e., temporally modulated waveforms^13^. Moreover, the high-level neocortical processing of vibrotactile and auditory input is done in close proximity^14^, with secondary auditory areas even responding to vibrotactile stimulation^15,16^.

Supporting speech intelligibility through vibrations is, nonetheless, challenged by the fact that skin mechanoreceptors that transduce vibratory signals are not ideal carriers of speech information. The frequency spectrum of perceptible vibrations only corresponds to that of low-frequency (< 700 Hz) speech signals^17^, whereas speech signals cover a wide band from 1000 to 7000–8000 Hz^18^. Consequently, the speech signal cannot be transmitted *as such* to the skin, as this would waste most of its energy. Several approaches have, thus, been employed to generate fully perceivable speech-derived vibrotactile stimuli to support SiN perception. A first one consists in the low-pass filtering of the speech signal such that the vibrotactile signal encompasses frequencies solely in the perceptible vibrotactile range. Drullman and colleagues^10^, for example, presented speech low-pass filtered at 200 Hz as a vibrotactile support, revealing significantly improved SiN perception. Alternatively, certain key features of speech signals can be extracted and utilized for generating the tactile input. Variations in pitch, for instance, can be conveyed by the changes in a speaker’s voice fundamental frequency (F0). Vibrotactile stimuli derived from the speaker’s F0 have been found to improve SiN perception in cochlear implant users^19^, as well as in simulated cochlear implant listening in normal-hearing subjects^6,9^. Another speech feature that has led to improved SiN comprehension when presented haptically in both cochlear implant^20^ and non-cochlear implant users^8^ is the low frequency (i.e., < 50 Hz) amplitude fluctuations over time, described by the speech temporal envelope. In contrast with F0 variations, the speech temporal envelope conveys more faithfully the variations in rhythmic structure, marking linguistic segments and prosodic content^21^. These findings evidently point towards a beneficial role of speech-derived vibrotactile input in SiN settings.

A range of aspects, however, remain to be addressed to better characterize the processes subtending the SiN perception enhancement afforded by vibrotactile stimulation. A major one pertains to the ability of normal-hearing listeners to innately (i.e., without training) benefit from added speech-derived vibrotactile stimulation in an ecologically valid SiN context. To the best of our knowledge, no study has utilized speech-derived tactile input to enhance SiN recognition of intact, non-vocoded speech presented in “*cocktail-party”* settings. For such multi-talker environments, access to temporal envelope information, rather than spectral information, may play a key role in segregating the speech stream of interest^22^, even when presented haptically. Temporal envelope-based tactile stimulation could provide cues about the temporal regularities of the speech stream of interest, aid prediction about upcoming speech input^23^, as well as enhance attentional processes to segregate the attended speech stream from the multi-talker background^24^. In untrained listeners, presenting a visual analog of the speech temporal envelope has already been proven to aid intelligibility of intact speech in multi-talker noise^25^, and a similar effect might emerge with the tactile modality. Another aspect concerning previously demonstrated vibrotactile SiN enhancement is the choice of the stimulation site. In audio-tactile SiN studies, this aspect is generally justified by attempts to maximize sensitivity (e.g., stimulation applied to the fingertips)^19^ or usability for potential real-world applications (e.g., stimulation applied to the wrists)^20^. In unimanual stimulation settings, however, the selection of either the dominant^5–7, 9^ or non-dominant^19^ hand as the stimulation location has not been justified, or not mentioned at all^10^. This comes into striking contrast with studies showing a greater involvement of the right hemisphere during audio-visual and audio-tactile integration^26,27^, suggesting a potential advantage of left-sided stimulation for eliciting larger multisensorial effects. Additionally, redundancy of perceptual input, such as exposure to simultaneous dual tactile stimuli, has been shown to improve perception^28^. Thus, bimanual stimulation with the same vibrotactile signal might lead to even greater multisensory benefits for audio-tactile SiN recognition in comparison with unimanual stimulation conditions. Lastly, hand vibrotactile perception sensitivity, typically evaluated using vibrotactile detection thresholds (VTs), varies in normal populations due to both inter-^29^, and intra-individual factors^30,31^. When thresholds were measured in previous studies, they were only used as a marker for normal vibrotactile perception^8,20^. The naturally occurring variation of tactile sensitivity could potentially influence the aiding effect of speech-derived tactile input at the time of testing, but this has not yet been assessed.

The present study therefore investigated the above-mentioned aspects by evaluating the speech recognition thresholds (SRTs) of normal-hearing listeners in a multi-talker background in conditions with (audio-tactile) and without (audio-only) temporal envelope-derived vibrotactile stimulation provided to the palms. This SRT measurement enabled us to measure the signal-to-noise ratio (SNR, in dB) at which a 50% understanding of a sentence occurs. The evaluation of SRT values was done in four experimental conditions: audio-only (AO), audio-tactile (AT) with left-hand stimulation (AT_left_), AT with right-hand stimulation (AT_right_) and AT with bimanual stimulation (AT_bilat_). Participants’ VTs were also measured for each palm. Under this framework, we specifically aimed to (i) determine if vibrations based on the speech temporal envelope aid SiN intelligibility in listeners without prior training, (ii) assess the effect of the chosen hand for stimulation, and (iii) investigate whether individual VTs can explain, in part, the ability to benefit from such additional speech-derived tactile cues. We hypothesized that SiN performance would be improved across all AT conditions compared to the AO condition. We also expected the greatest enhancement among the AT conditions to be brought on by bimanual stimulation, and between unimanual conditions, by left-hand stimulation. In addition, we anticipated that individuals with lower (i.e., better) VTs would benefit more from the concurrent vibrotactile input. As the VTs were measured per hand, we further hypothesized that higher vibrotactile sensitivity in one hand compared with the other (i.e., lower VT) would predict an enhanced ability to benefit from vibrotactile stimulation on that respective side.

## Results

**Figure 1:**
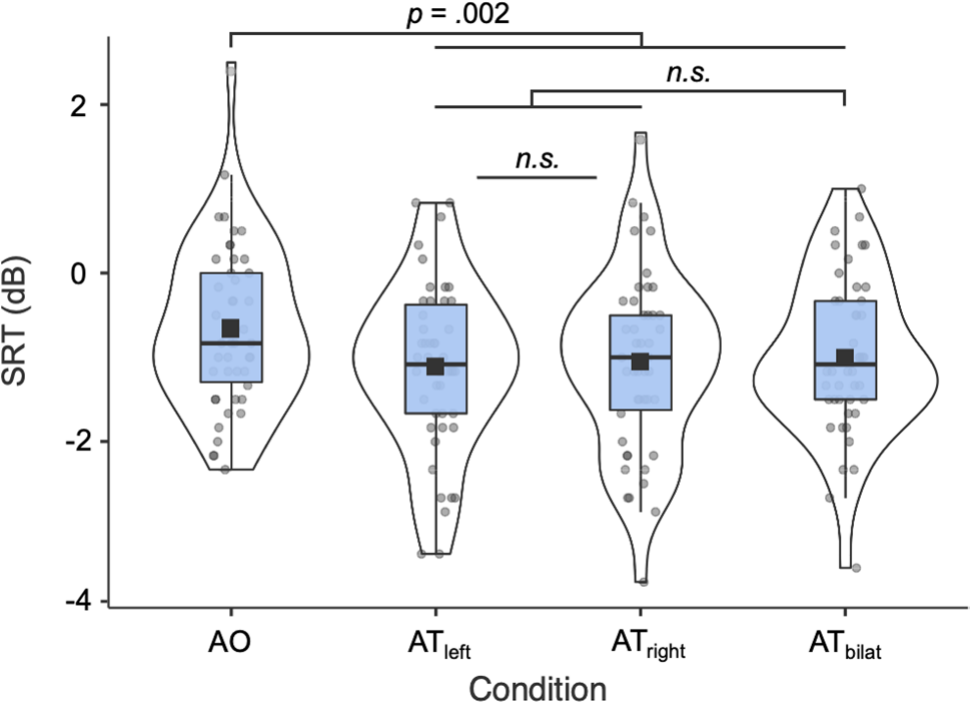
Impact of the experimental condition on speech recognition threshold (SRT) values. Violin plots of SRTs per condition with overlayed box plots. Single dots represent single participant data points. Horizontal line in violin represents the median, square represents the mean. AO, audio-only condition; AT_left_, audio-tactile condition with vibrotactile stimulation of the left hand; AT_right_, audio-tactile condition with vibrotactile stimulation of the right hand; AT_bilat_, audio-tactile condition with vibrotactile stimulation of both hands; n.s., not significant.

Figure 1 presents the distribution of average and individual SRT values in AO and AT conditions (AT_left_, AT_right_, AT_bilat_), with lower SRTs denoting better SiN recognition. In the AO condition participants had a mean SRT value of − 0.65 dB (*SD* = 0.98), meaning that, on average, participants could understand approximately 50% of a sentence when the SNR between the attended speech and the multi-talker noise was − 0.65 dB. SRTs were improved (i.e., lowered) by vibrotactile stimulation, with a mean SRT across AT conditions of − 1.06 dB (*SD* = 0.80). Mean SRTs were − 1.12 (*SD* = 1.01), − 1.07 (*SD* = 1.07), and − 0.99 (*SD* = 0.93) dB, for AT_left_, AT_right_, and AT_bilat_, respectively. At an individual level, 39 out of 46 participants had better SiN performance in at least one of the AT conditions compared to the AO condition, 32 in at least two out of the three, and 16 in all three. AT improvements in SRTs ranged between 0.17 and 4 dB (mean 1.06 dB, *SD* = 0.87), while the individual detriments (i.e., reduction in performance with added stimulation) were smaller, ranging between 0.17 and 2.2 dB (mean 0.67 dB, *SD* = 0.47).

A linear mixed-effect modelling (LMM) analysis was first conducted to evaluate the fixed effect of the experimental condition on the SRT values, with results revealing a significant effect (*F*(3, 138) = 3.59, *p* = 0.015, partial ƞ^2^ = 0.07). This indicated that SRTs were significantly influenced by the additional tactile stimulation. To evaluate the specific hypotheses concerning between-condition differences, planned Helmert contrasts were used. SRT values were significantly better (lower) across the pooled (i.e., averaged) AT conditions compared with the SRTs of the AO condition (mean improvement estimate = 0.41 dB, *SE* = 0.13 dB, *t*(138) = 3.18, *p* = 0.002, *d* = 0.54), indicating better SiN comprehension with additional haptic stimulation independently of the stimulation location. Bimanual stimulation did not yield a better enhancement compared to unimanual conditions, with AT_bilat_ SRTs not differing significantly from the averaged unimanual ones (*t*(138) = 0.75, *p* = 0.45). Lastly, no significant difference was found between the SRT values for AT_left_ and AT_right_ (*t*(138) = − 0.34, *p* = 0.73).

We then further sought to identify more clearly the factors influencing the extent of haptic improvement in the three AT conditions compared with the AO condition. For this purpose, an AT benefit score (in dB) was calculated for each AT condition as the difference between the AT SRT value and the AO SRT, using the formula: AT benefit = SRT_AO_–SRT_AT_. Positive AT benefit scores translate to better SiN performance in the AT condition. A second LMM analysis was performed to test how this AT benefit score varied based on the fixed factors of AT condition (AT_left_, AT_right_, AT_bilat_) and the SRT in the AO condition. AO SRTs were included as a fixed effect because poor SiN perception in the absence of any supplementary tactile cues was expected to correspond to an increased AT benefit, as shown in previous studies of haptic speech enhancement^6,9^. Two measures of vibrotactile perception were also included as fixed factors: the average vibrotactile sensitivity, calculated as the mean of both hands’ VTs, and the left–right vibrotactile sensitivity, obtained by subtracting the VT of the right hand from that of the left. Prior to running this analysis, the measured VTs were compared between hands with paired Wilcoxon signed-rank testing. While, on average, the left hand had lower VTs than the right (mean 0.150, *SD* = 0.12 and 0.174, *SD* = 0.12, respectively), this difference was not significant (*z* = 334, *p* = 0.06). Subjects with missing or extreme (± 3 *SD* away from the mean) VTs were not considered (*n* = 2). Interactions between the AT condition and the two vibrotactile sensitivity measures were also evaluated in the model.

**Figure 2:**
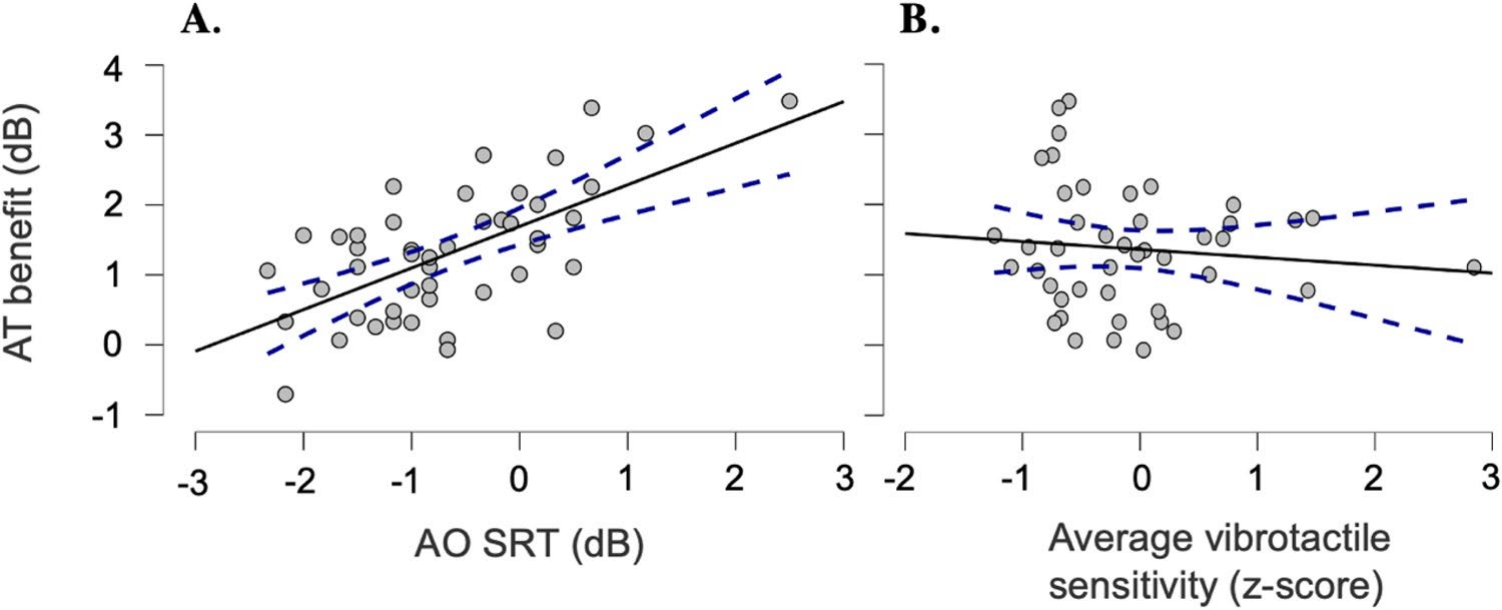
Significant predictors of audio-tactile (AT) benefit (in dB). Scatterplots indicating the relationship between AT benefit the and (**A**) speech recognition thresholds (SRT) in the audio-only (AO) condition and (**B**) average vibrotactile sensitivity (mean of the two vibrotactile thresholds—see *Methods*). Dotted lines correspond to the 95% confidence intervals.

The effect of each factor of interest on AT benefit scores is presented in Table 1. The analysis confirmed that the three AT conditions did not yield significantly different AT benefits (*F*(2, 88) = 0.44, *p* = 0.65), in accordance with the LMM analysis of the SRT values. As anticipated, an effect of AO SiN performance was identified (*F*(1, 44) = 38.07, *p* < 0.001, partial ƞ^2^ = 0.46), with poorer AO SRTs determining larger enhancements in speech comprehension with additional tactile support (*β* = 0.67, *SE* = 0.11, 95% CI = [0.44, 0.89]), Fig. 2A). The average vibrotactile sensitivity also had a significant effect on AT benefit (*F*(1, 44) = 6.79, *p* = 0.012, partial ƞ^2^ = 0.13), with higher (i.e., poorer) average VTs predicting a smaller AT benefit (*β* = − 0.27, *SE* = 0.10, 95% CI = [− 0.47, -0.07], Fig. 2B). Contrastingly, the left–right difference in VTs did not have a significant effect on the gained AT benefit (*F*(1, 44) = 0.51, *p* = 0.48). Lastly, the two-way interactions between the stimulation locations and the two vibrotactile sensitivity measures were not statistically significant (average vibrotactile sensitivity, *p* = 0.23; left–right vibrotactile sensitivity, *p* = 0.11).

**Table 1:** Linear mixed effects model for audio-tactile (AT) benefit.

Fixed effects	Num df	Den df	*F*-value	*p*-value
AO SRT	1	44	38.07	< .001
Average vibrotactile sensitivity	1	44	6.79	0.012
Left–right vibrotactile sensitivity	1	44	0.51	0.48
AT condition	2	88	0.44	0.65
AT condition * Average vibrotactile sensitivity	2	88	0.96	0.23
AT condition * Left–right vibrotactile sensitivity	2	88	2.98	0.11

AT benefit scores were used as the dependent variable, with the audio-only speech recognition threshold (SRT AO), average vibrotactile sensitivity (computed as average of both hands’ vibrotactile thresholds, VTs), left–right vibrotactile sensitivity (computed as difference between left and right VTs), AT condition (with levels left, right, and bilateral) added as fixed factors. Subjects were included as random effects. Num, numerator; Den, denominator; df, degrees of freedom.

## Discussion

This study demonstrates that the presentation of speech-derived vibrotactile cues significantly improves the understanding of intact (i.e., non-vocoded) speech in a multi-talker background in untrained listeners. This effect is observed irrespective of whether the vibrotactile stimulation is delivered to the left, right, or both palms. Moreover, this AT benefit increased with decreasing speech perception in noise performance and with increasing vibrotactile sensitivity. 

The key finding of this study is that vibrotactile stimulation based on the speech temporal envelope robustly supports SiN recognition of untrained, normal-hearing subjects in a multi-talker background. Vibrations significantly lowered SRTs by up to 4 dB (0.41 dB on average) compared to AO conditions. Typically, haptic enhancement of speech perception in subjects with normal hearing ability is observed after substantial a priori exposure to coupled AT stimuli during training sessions^8,9^. In contrast, our experiment demonstrated a significant, albeit modest, haptic enhancing effect in a group of normal-hearing participants without any prior training. A limited number of other research works also aimed to enhance SiN recognition in untrained subjects using tactile cues derived from the speech temporal envelope but did not uncover significant effects. One such investigation delivered speech envelope-based vibrations to participants’ right index finger during simulated cochlear implant listening in multi-talker conditions^8^. A statistically nonsignificant improvement of 5.4% in the percentage of keywords correctly identified was found in the AT condition compared with the AO condition in the absence of previous training. Another study^32^ supplemented degraded speech with envelope-based vibrations delivered bimanually, and also found a nonsignificant average improvement in intelligibility of only 1.2% of words identified in noise. The current study did not allow for an equivalent evaluation of the effect of tactile stimulation in %, as the obtained SRT for each experimental condition was always associated with a 50% speech understanding. However, based on previous research^10^, the uncovered AT benefit in dB could be equated to a 3–4% improvement, which would be consistent with the above-mentioned results. The lack of a statistically demonstrable effect in both previous studies^8,32^ may be due to the lower number of participants (i.e., 8 and 18, respectively) and the small extent of this enhancement in untrained subjects. Our cohort, which included a substantially larger group (*n* = 46), allowed for a more robust detection of the hypothesized effect. It is nevertheless difficult to directly compare results between studies, due to differences with regards to the used speech corpora, as well as the methodology for evaluating SiN intelligibility (using word scoring as opposed to sentence scoring). Of note, the measured AO SRTs of the current study are relatively poorer than those generally observed in SiN multi-talker conditions^6^, which could be due to either the SRT testing conditions or stimuli generation procedures. Therefore, future replication studies are warranted.

A series of other works found ampler benefits of speech-derived vibrotactile stimulation for SiN understanding, ranging from 2.2 to 6 dB, even without training^6,19^. However, in these studies, the listeners were either cochlear implant users^19^, or the target speech signals were in a non-native language and vocoded^6^. Such scenarios provide especially challenging conditions for understanding SiN, leaving more space for speech-derived tactile input to support speech perception. This reasoning is in line with the *inverse effectiveness principle*^33,34^, which stipulates that in settings where unimodal performance (here, auditory) is impaired or reduced, congruent (i.e., synchronous) multisensory cues (here, tactile) are more likely to be integrated to enhance perception. Moreover, for hearing-impaired listeners, as tested in^19^, neuroplastic changes whereby haptic input can be processed in auditory cortex^35^ might augment AT integration capacity. The present study tested a cohort of healthy participants using intact speech embedded in unaltered multi-talker background noise, resembling a situation which could be encountered in everyday life. As such, these conditions did not fully conform to conditions that would potentate the inverse effectiveness principle, leading to small multisensory benefits^33^. Still, it could be observed that participants who benefited the most from the additional vibrotactile stimulation were the ones who had poorer SiN performance in a unisensory (AO) setting. For future studies on normal-hearing subjects, the use of vocoded speech in conjunction with measurements of SRTs corresponding to intelligibility levels lower than 50% might create conditions conducive to an increased AT benefit.

With regards to the effect of the chosen hand for stimulation, contrary to the expected superiority of bimanual over unimanual AT stimulation, and of the left- over right-hand stimulation, results indicate a similar enhancement of SiN recognition across all three AT conditions. Our initial assumption regarding bimanual stimulation was based on the expected cumulative effects of providing redundant (i.e., identical) tactile input to both hands^28^. While participants did indeed report that they perceived more strongly bimanual rather than unimanual stimulation, this did not reflect on the SRT values or the AT benefit scores. For the unimanual conditions, the hypothesized left-side advantage was based on previous works reporting a stronger involvement of the right hemisphere during other multisensory tasks involving the auditory modality^26,27^. Nonetheless, these studies investigated simpler auditory tasks, such as auditory sensitivity and detection, not speech processing per se. Early, sublexical stages of speech perception are, notably, achieved bilaterally^36^. It is thus difficult to anticipate the laterality (or lack thereof) during speech-specific audio-tactile interactions. Despite contradicting our initial hypothesis, our results coincide with the AT benefit uncovered in prior works during SiN tasks with left^19^, right^6,9^ and bimanual^20^ tactile stimulation. The comparable behavioural enhancement across the AT conditions is suggestive of a common underlying neural mechanism. Somatosensory stimulation has been found to directly modulate the ongoing cortical activity of the auditory cortices through a phase-resetting process^37^. Vibrotactile signals, in particular, elicit early (i.e., within 100–200 ms from the stimulus presentation) activity in both ipsi- and contralateral secondary somatosensory cortices and superior temporal gyrus, which encompasses the auditory association cortex^15,38^. This early convergence of vibrotactile input onto bilateral auditory regions may explain the possible route through which the AT modulation of speech recognition takes place.

Another main finding of the present study is that the extent of the AT benefit is significantly predicted by average vibrotactile sensitivity levels (i.e., averaged VTs of the two palms). Participants with lower VTs, translating to higher sensitivity levels, gained larger AT benefits from the additional temporal envelope-based vibrations. Results nevertheless contradict our expectation of a side-specific enhancement where, for example, better right-hand sensitivity corresponding to a smaller VT in the right than left hand, would indicate greater AT_right_ benefit: no significant interaction was found between the left–right difference in VTs and the stimulation location. Rather, results point towards a general vibrotactile sensitivity capacity that can modulate the enhancement brought on by speech-derived tactile stimulation. This is partly confirmed by other research works aiming to haptically improve SiN perception conducted in participants with ample age ranges^19,20^, which show highly extensive inter-individual variation in the observed AT benefit. We argue that the inter-individual variability in vibrotactile sensitivity could partially explain such findings, given its known variation in relation to aging^29^. Future research should implement vibrotactile detection threshold testing procedures to better predict the multisensory gain afforded by haptic input during SiN perception. Measurement of VTs could also allow for individualized stimulation intensity levels, to maximize AT enhancement of SiN recognition.

Generally, ampler multisensory benefits during SiN perception are afforded by visual speech cues, ranging from 4 to 15 dB^2–4^. The emergence of a robust *haptic* enhancing effect in untrained, normal-hearing listeners and in the adverse but ecologically valid auditory conditions employed in the present study is indicative of the propensity to exploit non-auditory cues during SiN recognition. This, in turn, provides support to the multimodal view of speech perception^39,40^. According to multimodal speech theories, speech perception can be shaped by any non-auditory stimuli, provided that these contain *modality-neutral* speech information reflective of the same speech event. Temporal envelope-based cues extend across larger linguistic units, highlighting the intrinsic rhythmicity of the auditory speech signal, by marking the onset and offset of syllables, words and phrases^41^. As such, it is a time-varying speech feature which can potentially be instantiated auditorily, haptically, and even visually. Indeed, the presentation of a more abstract visual equivalent of the speech temporal envelope in multi-talker background noise conditions provides an improvement in speech intelligibility accuracy of approximately 7%^25^ compared to AO conditions. The estimated 3–4% improvement uncovered in our study suggests that both the tactile and visual systems can be utilized as transmission channels for speech-related cues. This has been confirmed by Oh et al.^42^ who evaluated SRTs in speech-shaped noise of untrained subjects using either a visual or a tactile instantiation of the temporal envelope of the attended speech stream. The enhancement in dB was not significantly different between the two modalities, with both providing an enhancement from AO conditions of approximately 2 dB. As such, the increased benefit of typical visual speech cues (i.e., lip movements) could be attributable to the continuous encoding during our lifetimes of speech-related audio-visual content, rather than to a primacy of visual input over tactile. Future studies comparing abstract instantiations of the same audio-tactile and audio-visual speech feature could reveal similar multisensory benefits in multi-talker listening conditions.

An alternative—but not exclusive—perspective on the observed beneficial effect of tactile input on SiN perception is that speech-derived tactile stimulation does not lead to true multisensory integration, but rather an attentional-level enhancement. This possibility is supported by the type of cues that vibrations derived from speech temporal envelope provide, allowing for the identification of voiced speech (i.e., the moments when the speaker is talking). Moreover, in multi-talker settings, top-down attention has been identified as a key factor in attending to the speech stream of interest^43^. The speech-derived vibrations used in the current study could have informed participants about when the main speaker started to speak, as well as make them inadvertently more attentive than in AO conditions. Previous research on audio-tactile SiN recognition has found a similar enhancement brought on by congruent and incongruent (i.e., of a different sentence) vibrotactile stimuli, which would support the attentional, rather than true integratory, perspective^9^. To better delineate attentional effects, upcoming studies could utilize eye-tracking technology for the online evaluation of attention levels during SiN recognition in AT and AO conditions through pupillary dynamics^44,45^, both with matching and non-matching vibrotactile input with the same onset as the target speech.

Overall, these results strengthen the cross-modal viewpoint of speech perception and demonstrate the remarkable potential of somatosensory input, as opposed to the classically acknowledged visual one, to support SiN perception. Although the haptic enhancement in SiN performance of the present study is modest, the uncovered inverse relationship between AO SiN performance and AT benefit is highly suggestive of larger benefits for populations with impaired comprehension under adverse auditory conditions, as per the inverse effectiveness principle^33^. Cochlear implant users, specifically, have difficulties perceiving low-frequency cues such as speech temporal envelope information and segregating sounds in SiN scenarios^19^. Hence, the current results reinforce the potential of the usage of haptic input derived from speech temporal envelope for this category of users, in whom training regimes have already revealed robust improvement of SiN performance^20^. Furthermore, the presence of this beneficial effect in naïve (i.e., untrained), normal-hearing participants, expands the range of users that could benefit from supplemental speech-derived tactile input for better SiN recognition. These could include patients with SiN perception difficulties despite normal peripheral hearing function^46^ and even individuals with attention deficit disorders. For the latter, the selective attendance of one speaker in a multi-talker background is especially attentionally demanding^47^ and supplementing this process through the delivery of envelope-based tactile stimulation is an interesting and underexplored avenue of research. Lastly, these findings indicate that the extent of AT enhancement positively depends on individual vibrotactile sensitivity levels, evaluated using detection thresholds. As such, individually adapted intensity levels of speech-derived supplemental haptic input should be considered in the design of haptic assistive communication devices aimed at improving SiN perception.

## Materials and methods

### Participants

Forty-six native French-speaking adult participants (18–33 years, 34 females, mean age = 20.3, *SD* = 2.86) were included in the study. They were right-handed (mean score of 86, and *SD* of 18.7 on a laterality quotient scale from − 100 to 100) as evaluated using the short version (7-item) of the Edinburgh Handedness Inventory^48–50^. All had normal hearing as indicated by air-conducted hearing thresholds of ≤ 25 dB HL (hearing level) during pure-tone audiometric testing at each of the standard frequencies between 125 and 8000 Hz. Three additional participants were tested but not considered in the analyses as they had average SRT scores above three standard deviations from the mean performance. All participants completed a short screening questionnaire where they could report whether they were on any medication or had any known psychiatric, auditory, neurological, or somatosensory medical condition, with none indicating any known pathologies. All participants were students of the *Université libre de Bruxelles*, gave informed consent and received course credits for participating in the study. The study was approved by the Ethical Committee of the ULB—Hôpital Erasme (P2012/049) and performed in accordance with the approved guidelines and regulations.

### Stimuli preparation

#### Audio stimuli

The speech material was taken from the French Sentence Test for Speech Intelligibility in Noise (FIST)^51^ for European Francophone listeners. FIST provides a male sentence corpus consisting of 20 training sentences and 14 testing sets of 10 sentences each with an average length of 3.02 s and a mean syllable count of 10. All FIST sentences are spoken by the same speaker and matched for difficulty, naturalness, content, level of abstraction, and intelligibility in noise^52,53^. For the SiN testing and the practice session, a 5-min multi-talker noise composed of the speech of 3 females and 3 males was used^54^, with none of the male speakers corresponding to the FIST corpus speaker. This type of background noise can provide both energetic (i.e., peripheral) auditory masking and lexical (i.e., central) interference^55,56^, without being as attentionally demanding as multi-talker noise with fewer speakers^57^. Speech-on-speech masking also provides a complex, yet ecologically valid, auditory scenario where, according to the principle of inverse effectiveness, multisensory integration should be boosted^33^. After a root mean square normalization procedure, FIST sentences were mixed with random 7-s excerpts of the multi-talker noise at SNRs ranging from − 10 dB to 10 dB, in intervals of 1, so that they were readily available during the testing procedure. The SNR was modified by varying the level of the speech signal. All the final auditory stimuli had a total length of 7 s and started with the multi-talker background and ended once the FIST sentence ended.

#### Tactile stimuli

**Figure 3:**
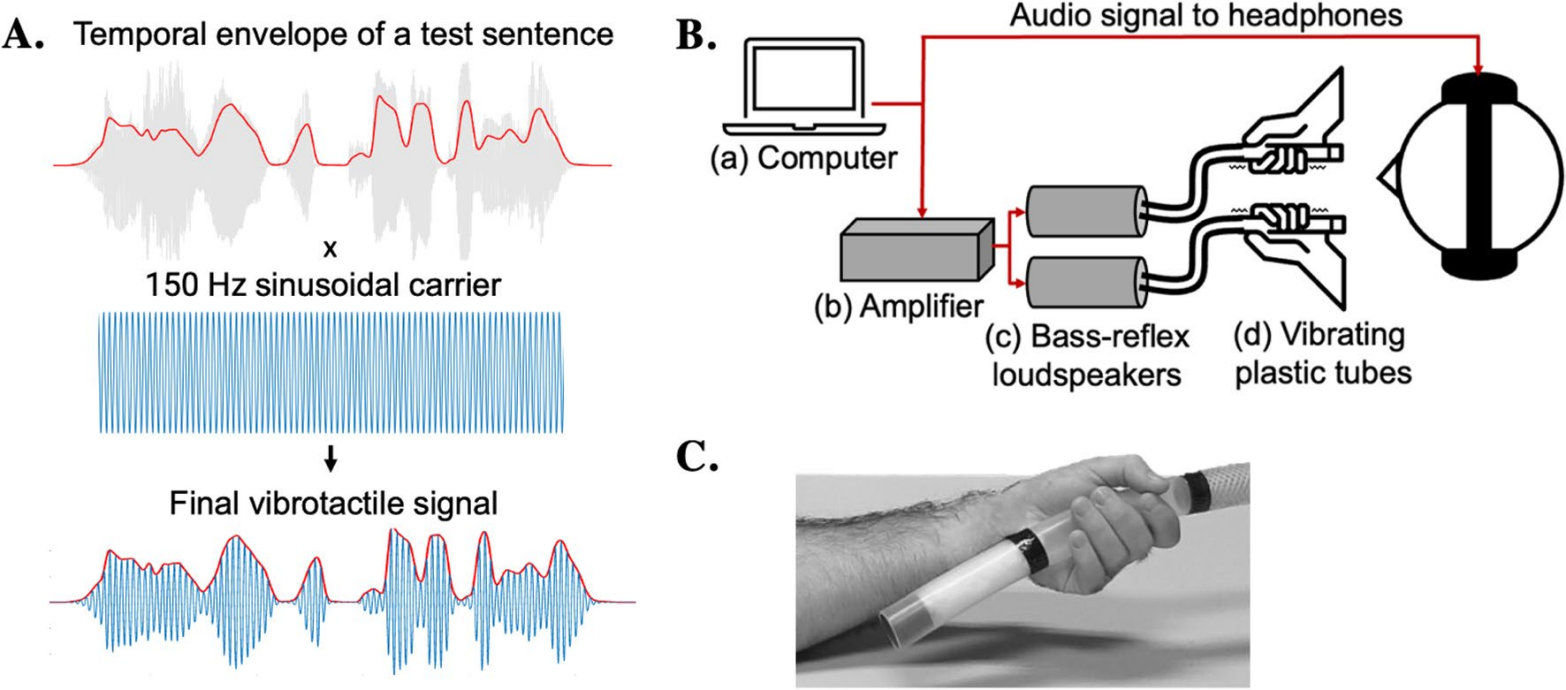
Vibrotactile signal generation and experimental setup. (**A**) Speech temporal envelopes (red line) of FIST sentences were extracted (here, the envelope corresponds to the sentence “Mon chien est très intelligent”) and combined with a 150 Hz sinusoidal wave, such that the final vibrotactile signal (bottom left figure, in blue) had a frequency content around 150 Hz with amplitude variations (in red) as per the speech temporal envelope. (**B**) A main computer (a) was used to simultaneously deliver the vibrotactile and auditory stimulation. The vibrotactile signals were first passed through an amplifier (b) and two bass-reflex speakers (c), one for each hand. The vibrations reached participants’ palms through two plastic tubes (d) with vibrating silicone endings. (**C**) Participants held the tubes between two marked lines (black lines).

To obtain the vibrotactile stimuli derived from the temporal envelope of the attended speech stream, the temporal envelopes of all FIST sentences were first extracted using a previously established approach^58–60^. This entailed passing each audio signal through a gammatone filter bank with 31 equally spaced channels from 150 to 7000 Hz on the Mel scale, covering the ordinary frequency range of human auditory perception^17^ and of the average speech spectrum^18^. The Hilbert transform was then utilized to compute the temporal envelope of each channel of the filter bank, and their average was taken as the final speech temporal envelope. Figure 3A shows the temporal envelope of one of the used FIST sentences and the subsequent generation of the vibrotactile signal by using it to amplitude modulate a 150 Hz sinusoidal carrier. This tonal carrier frequency value was chosen by first considering the prime sensitivity range of vibratory Pacinian mechanoreceptors in humans (between 100 and 300 Hz)^61^. Following this, behavioural piloting (*n* = 5) was conducted to identify the carrier frequency producing the most subjectively potent sensation at a pre-set vibration magnitude. Briefly, participants listened to the same short (15 s) excerpt of speech accompanied by synchronous vibrotactile stimulation composed of randomly ordered 100 to 250 Hz sinusoidal carriers at 25 Hz intervals modulated by the speech temporal envelope. They were then asked to select the vibrotactile stimulation that they perceived as being the most intense. Four of the five subjects selected the 150 Hz stimulation that was thus chosen for the main experiment. Upon presentation, the vibrotactile stimulation was always congruent with the speech stream that participants had to attend to, both in the pilot and in the main experiment. This is in line with the temporal rule of multisensory integration^62^, which posits that sensorial events are more likely to be integrated if they take place simultaneously. The synchronicity between the two signals was verified using an accelerometer prior to the experiment. All signal processing and generation of speech and speech-derived vibratory stimuli were done in Matlab (R2021a, TheMathWorks).

### Apparatus

The experimental setup is illustrated in Fig. 3B. A main computer controlled the delivery of the stimuli using custom-designed Python3 scripts (Python Software Foundation, https://www.python.org/). The auditory stimuli were presented diotically through Soundcore Life Q30 noise-cancelling headphones at approximately 65 dB SPL (sound pressure level), as measured using a sound level meter (Sphynx Audio System) and kept at the same volume intensity for all participants. The vibrotactile stimulation set-up was similar to that used in Caetano and Jousmäki^15^ and other studies^63,64^. In practice, vibratory stimuli were delivered through two blind-ended rigid plastic tubes (⌀38 mm, 3-m long) attached to two bass-reflex loudspeakers with coaxial elements connected to an amplifier (t.amp TA 2400 MK-X). The participant-held endings of the tubes (Fig. 3C) were made from a more flexible, vibratory-purposed silicone that had sound-attenuating material (cotton wool) at the closed end. The vibrating area was limited to a palm-sized region, which was visually marked. Participants were instructed to respect the limits of the markings when holding the tubes. The two tubes were partially fixated to the table such that they could be held almost passively, without exerting too much force. To limit any potential discomfort, subjects were given cushion-like arm rests. Although the two plastic tubes were designed to be as similar as possible, to potentially counteract any effect of tube characteristics, we reversed their position (i.e., the tube which was used for the left hand was used instead for the right, and the same for the other one) for half of the participants. A brief post-testing analysis further revealed that this position did not have a significant effect on SRT values in any of the experimental conditions (mean SRT difference of 0.09, 0.06, and 0.1 for AT_left_, AT_right_, and AT_bilat_, all *p* > 0.5; independent t-tests).

Amplifier settings were kept constant across participants during the SRT evaluation. The vibration intensity was set to a fixed magnitude that was clearly perceptible in all participants during the behavioural piloting (*n* = 5) and low enough not to lead to any auditory percept. During the VT measurements, the intensity was reduced by approximately 10 dB to include levels at which it did not produce any discernible tactile percept, as tested in three subjects that did not participate in the main experiment.

### Procedure

Participants were seated in front of a table in a quiet experimental room. The experimenter was present in the same room and controlled the computer that was delivering the stimuli, with no direct line of sight to the participant. The experiment began with asking the participants to complete a brief screening questionnaire. After this, the audiometric hearing thresholds were measured using a MADSEN Xeta audiometer (MADSEN, GN Otometrics, Denmark).

#### Vibrotactile detection threshold measurement

During the VT measurement procedure, the approximate lowest amplitude value at which participants could perceive a 150 Hz (i.e., the frequency used to deliver speech envelope stimulation) vibration was identified. Participants were first guided by the experimenter on how to properly position their hands such that the inner surface of palm touched the tube. Then, they were instructed to verbally indicate whenever they felt or not a vibration after hearing a 200-ms 500 Hz beep in their headphones. To limit the sound of the stimulation set-up, which could potentially act as a cue for detecting the vibrations, a pink noise masker^54^ was concurrently presented in the headphones for the whole duration of the threshold measurements. Thresholds were determined using a ‘yes–no’ detection task^65^ following an adaptive staircase (i.e., up and down) procedure^66^, with the amplitude of the vibrations varied by modifying the voltage output of the computer’s audio connector. In practice, vibrations were presented at amplitudes that alternated between high and low values. Initially, vibrations for the high values were clearly perceptible, and those for the low values were imperceptible. The interval (i.e., step size) between the high and low amplitude values was then gradually decreased, until it converged towards an approximate threshold level. The final VT value was calculated as the average of the final 4 amplitudes presented to the participant. Lower VT values reflected higher sensitivity levels, i.e., the ability to detect a smaller displacement of the tube. The VT measurement procedure conforms to the recommendations specified by international standards^67^ regarding the frequency of the tested vibration, vibrometer components and psychophysical algorithm choice.

For data analysis, two vibrotactile sensitivity measures were computed using the measured VTs: an average vibrotactile sensitivity score by averaging the VTs over the two hands, as well as a left–right vibrotactile sensitivity score by subtracting the VT of the right hand from that of the left. The latter indicated whether the more sensitive hand was the left (i.e., through a negative value) or right one (i.e., through a positive value), and allowed for a testing of the interaction between this hand advantage and the AT condition.

Following the VT measurement, participants were asked to continue holding the vibrating tubes and attend to an audio recording (without added noise) for approximately 6 min (mean ± *SD*, 6.0 ± 0.3 min) accompanied by its corresponding vibrations derived from the speech temporal envelope. This was intended to briefly familiarize participants with the tactile stimulation. During this session, the vibration was presented twice to either the left, right or both hands, in random order and in blocks of 1 min (mean ± *SD*, 58.8 s ± 3.4 s), totaling 2 min of exposure for each stimulation condition.

#### Speech-recognition threshold testing

Prior to the SRT testing, participants were told that they had to fully repeat sentences spoken by a male speaker against a multi-talker background. If unable to do so or unsure of what they had heard, they were instructed to still mention the parts of the sentence that they understood. To familiarize participants with the voice of the FIST speaker and the multi-talker background, they first practiced the testing procedure on the two training sets, with and without noise (i.e., the raw sentence material), respectively. This allowed for the identification of any participant with verbal working memory or hearing-in-noise issues, which would have not been detected during the audiometry. The testing continued with the SRT evaluation. The first sentence of a randomized FIST sentence set was presented at a SNR of 0 dB, after which a simple up-down adaptive procedure in 2 dB steps was employed, as per Plomp and Mimpen^68^ and typical adaptive hearing-in-noise testing procedures^69,70^. More specifically, if the sentence was correctly identified by the participant, the next one would be presented at a SNR 2 dB lower. If the participant could not correctly identify it, the following would be presented at a SNR 2 dB higher. This was done until the full set of sentences was presented to the participant. The sentence scoring was *verbatim,* meaning that all the words in it had to be repeated exactly for it to be considered understood^52^. A small number of exceptions were accepted, such as one-letter variations of definite or indefinite articles and pronouns or verb tense incongruencies. SRTs for each set were calculated as the mean of the SNRs of the last 6 sentences, and that of a hypothetical 11th^52^. For each of the four conditions (the AO and three AT conditions), SRTs were measured twice, leading to a total of 8 sentence lists being presented per participant. The assignment of conditions to sets from the pool of 14 FIST sets was random for each participant. The condition order was also randomized, with the restriction that the SRT could not be tested twice in a row for the same experimental condition.

For the data analysis, the two SRTs for each condition were averaged. To quantify the multisensory AT benefit brought on by the vibrotactile stimulation, SRTs were used to calculate an AT benefit score (in dB) in each participant, for each of the three AT conditions. This was done by subtracting the SRTs of the AT condition from that of the AO condition, as per the formula: AT benefit = SRT_AO_–SRT_AT_. Larger AT benefit values were indicative of larger enhancements with concurrent tactile input compared to AO conditions.

### Statistics

Linear mixed modelling (LMM) was used to evaluate the fixed effect of the vibrotactile stimulation on SiN performance (SRT values) (*n* = 46). A second LMM was conducted to analyze how AT benefit values depend on the stimulation location, average vibrotactile sensitivity (i.e., the mean of the VTs of the left and right hand), the left–right vibrotactile sensitivity (i.e., the difference between the two VTs) and AO SRT values (*n* = 44). Both LMMs had a by-subject random intercept. Residual normality for mixed modelling was assessed with no significant violation using both the Shapiro–Wilk and residual Q–Q plots. Degrees of freedom were estimated using the Satterwhite method. The limits of the 95% confidence intervals of beta coefficients were obtained using bootstrapping (R = 500). Parameter estimation was done using the maximum likelihood estimation method. Between-hand differences in VTs were evaluated using Wilcoxon signed-rank testing, while tube positioning effects on AT SRTs were tested using independent t-tests. Significance level was *p* = 0.05.

## Data Availability

The dataset analyzed in the current study can be obtained upon reasonable request to the corresponding authors.
